# Sleeve Gastrectomy Leads to Immediate, Significant Intraoperative Increase in Lower Esophageal Distensibility and Opening Area

**DOI:** 10.3390/jcm15020701

**Published:** 2026-01-15

**Authors:** Michael de Cillia, Christof Mittermair, Hannes Hoi, Martin Grünbart, Helmut Weiss

**Affiliations:** Department of Surgery, Saint John of God (SJOG) Hospital, 5020 Salzburg, Austriahannes.hoi@bbsalz.at (H.H.); chirurgie@bbsalz.at (H.W.)

**Keywords:** obesity, bariatric surgery, surgical innovation, sleeve gastrectomy, gastric bypass, distensibility index (DI), impedance planimetry, intraoperative diagnostic

## Abstract

**Background/Objectives:** Functional impairment of the complex motility system in the upper gastrointestinal tract is high in patients suffering from obesity and even higher after metabolic bariatric surgery (MBS). Sleeve gastrectomy (SG) and gastric bypass (GB) represent the most common MBS procedures worldwide. Despite procedural standardization, no diagnostic method is able to depict the functional consequences resulting from intraoperative anatomical changes during MBS. This pilot study was conducted to reveal immediate intraoperative functional effects of MBS on the anti-reflux barrier in SG and GB. **Methods:** A prospective analysis was performed on consecutive patients with informed consent for MBS. A standard protocol for each procedure was established prior to study onset to analyze functional parameters at the lower esophageal sphincter (LES). Measurements were conducted intraoperatively during minimally invasive SG and GB. Distensibility index (DI), intra-balloon pressure, diameter (Dmin), and minimal cross-sectional area (CSA) at the LES served as points of interest for analyzation. **Results:** Intraoperative evaluation was performed successfully in 40 patients and no directly related adverse events were reported. DI and Dmin intraoperatively significantly increased immediately in SG (2.1 mm^2^/mmHg (±0.5) vs. 2.9 mm^2^/mmHg (±1.3), 95% CI: −1.6 to −0.14, *p* = 0.023 and 12.0 mm (±1.2) vs. 13.9 mmH (±2.8), 95% CI: −3.6 to −0.2, *p* = 0.028, respectively) whereas GB did not affect functional measurements. **Conclusions:** Sleeve gastrectomy immediately and significantly influences the LES and increases the opening area whereas gastric bypass surgery appears not to influence LES distensibility or opening diameters. Intraoperative standardized EndoFLIP^TM^ measurements are feasible and safe and add additional real-time information during MBS.

## 1. Introduction

The upper gastrointestinal tract (UGIT) is a complex motility system of optimally aligned components. Disruption of this functional architecture can lead to a variety of disorders such as disturbed esophageal clearance in achalasia and eosinophilic esophagitis or gastroesophageal reflux disease [1,2,3,4]. The prevalence of functional gastrointestinal impairment is high in patients suffering from overweight and obesity, but it is acknowledged even more after metabolic bariatric surgery (MBS) when food restriction necessitates surgical modifications of the UGIT [5].

The sleeve gastrectomy (SG), one-anastomosis gastric bypass (OAGB), and Roux-en-Y gastric bypass (RYGB) are regarded as the main pillars of MBS. Over recent decades, numerous intraoperative techniques have evolved to standardize these surgical reconstructions, such as the use of diameter-calibrating catheters, defined staple line formation, or length measurements [6,7].

Until now none of these methods were able to depict the functional consequences resulting from intraoperative anatomical changes during MBS. Unrecognized high-pressure zones, dilations, or incorrect gastric sleeve formation carry a risk of postoperative adverse events such as reflux symptoms, staple line insufficiency, stenosis, or fistula formation. However, the chronology of adverse event development has not been fully elucidated. It remains uncertain whether its origin is directly linked to intraoperative procedure-specific factors or whether it emerges later in the postoperative course because of healing dynamics, scar formation, or patient-specific factors [6,7,8,9,10].

Recent years have seen increased scientific exploration and clinical application of the functional lumen imaging probe EndoFLIP^TM^ (Medtronic plc, Dublin, Ireland). This device has already provided scientific impact for a more detailed evaluation and understanding of dysfunction of the lower esophageal sphincter (LES), especially in the context of achalasia and reflux disease [11,12].

The goal of this pilot study was to assess if surgical creation of gastric sleeves and gastric pouches directly influence the architecture and functional conditions at the gastroesophageal junction.

## 2. Materials and Methods

The EndoFLIP^TM^ (Medtronic plc, Dublin, Ireland) technology is functionally based on impedance planimetry. It generates parameters from changes in diameter and intra-bag pressure that occur due to individual luminal geometry during the volume-controlled expansion of a catheter-based balloon that contains 16 electrodes immersed in saline solution [13]. This technique delivers the potential to concurrently visualize luminal geometry and gain a better understanding of dynamic processes at gastrointestinal sphincters and luminal reconstructions, even during the operative procedure. Particularly in reflux surgery, EndoFLIP^TM^ has been integrated into surgical practice, as it provides a stand-alone feature for measuring functional changes in real time occurring at the LES during an anti-reflux procedure, after establishing normative values for distensibility at the LES [14,15].

The study was officially registered in the German Clinical Trial Registry (DRKS-ID: DRKS00033317, date of registration: 26 January 2024). From May 2024 to October 2025, 40 patients signed informed consent and underwent minimally invasive MBS with intraoperative use of the EndoFLIP™ device at the Saint John of God Hospital, Salzburg, Austria. The use of EndoFLIP^TM^ was carried out in accordance with the intended purpose to evaluate dimensions and balloon pressure in the digestive tract. The definition of a study population size was not applied due to the pilot nature of the study.

**Figure 1:**
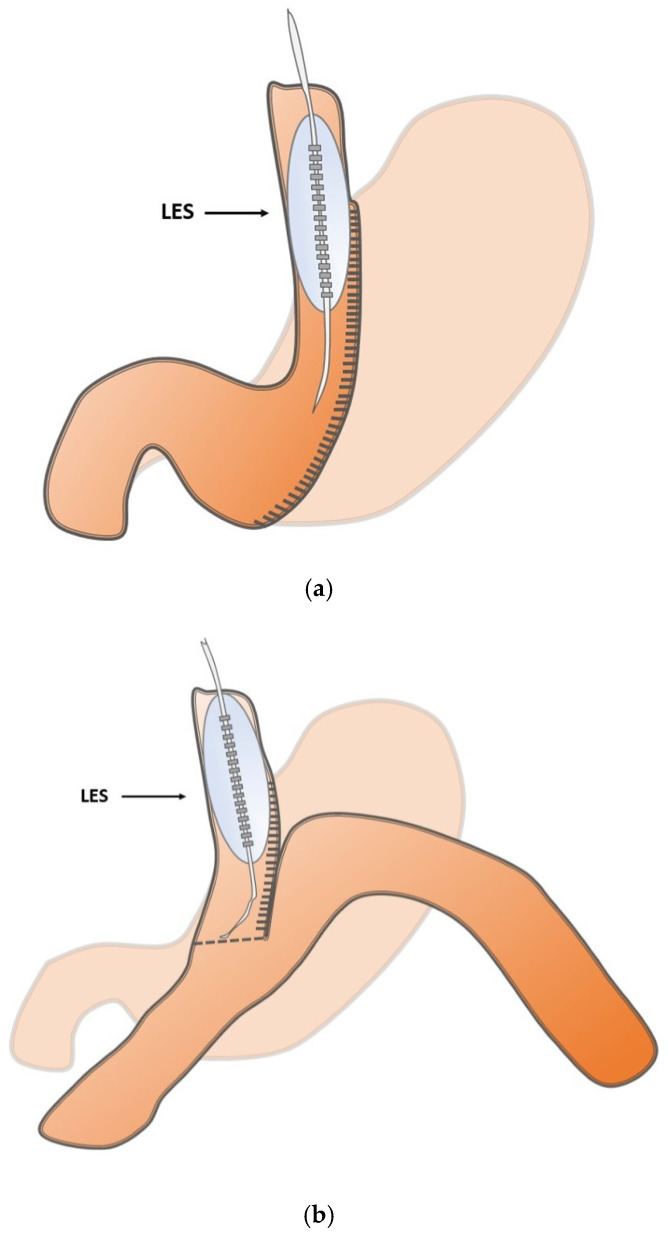

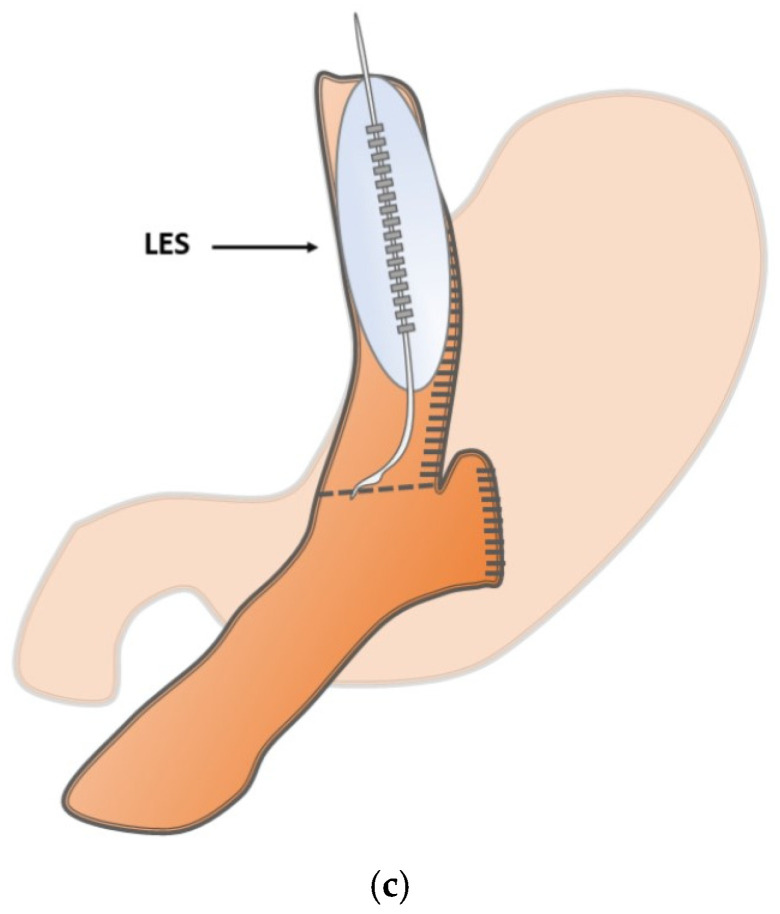
(**a**) Schematic illustration of the intraoperative measurement points in sleeve gastrectomy at the lower esophageal sphincter (LES). (**b**) Schematic illustration of the intraoperative measurement points in one-anastomosis gastric bypass at the lower esophageal sphincter (LES). (**c**) Schematic illustration of the intraoperative measurement points in Roux-en-Y gastric bypass at the lower esophageal sphincter (LES).

Inclusion criteria for this prospective observational pilot study were patients aged 18 or older who signed informed consent and were seen to have an interdisciplinary indication for MBS. Procedures comprised gastric sleeve resections, one-anastomosis gastric bypass, and Roux-en-Y gastric bypass. Revisional surgeries as well as patients with prior UGIT surgery or major esophageal motility disorders were excluded. The study protocol defined the LES as a point of interest in the respective reconstruction to play a pivotal functional role (Figure 1a–c).

The bypass group (OAGB and RYGB) were pooled because major technical differences are related to limb length and post-anastomotic anatomy, whereas the surgical modification at the defined measurement site (LES) is comparable, as shown in Figure 1b,c.

Sleeve formation and pouch creation followed standardized procedural steps using a 32 French gastric tube, as described elsewhere.

**Figure 2:**
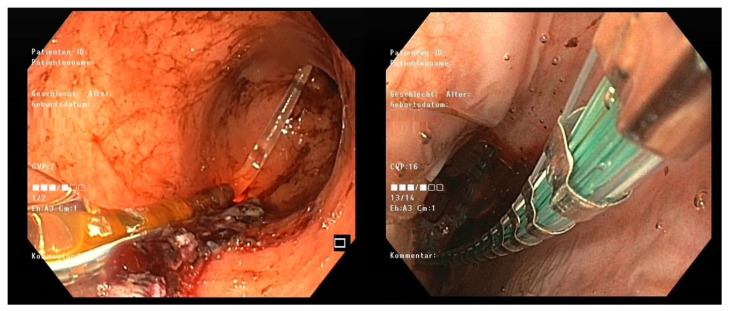
Endoscopic positioning of the EndoFLIP^TM^ balloon: visualizing the correctly positioned catheter tip in the body of the sleeve (**left**) and the balloon at the LES (**right**).

The 8 cm EF-325 EndoFLIP™ probe was applied intraoperatively by the same expert operator in all patients. In brief, this was accomplished intraoperatively as follows: the EndoFLIP™ was introduced transorally immediately after finalizing the gastric reconstruction or anastomosis in MBS. To ensure correct balloon placement at the defined section, laparoscopic identification of the balloon or transoral endoscopic guidance was mandatory to exclude direct balloon-related complications (Figure 2).

**Figure 3:**
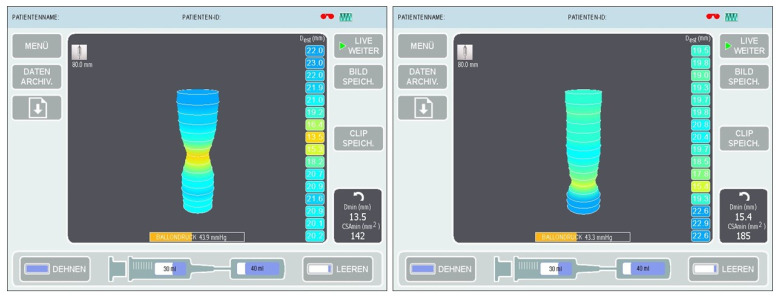
Digital planimetric images during intraoperative measurements at the lower esophageal sphincter (LES) in sleeve gastrectomy (**left**) and Roux-en-Y gastric bypass (**right**).

The balloon was then filled with saline to a volume of 40 mL and measurements were taken (Figure 3) at the expiratory endpoint in a 30° anti-Trendelenburg position after releasing the pneumoperitoneum with a calibration period of 30 s. The EndoFLIP™ was removed thereafter and an endoscopic air leak test was performed.

Primary outcome parameters were defined as immediate intraoperative changes in the EndoFLIP™-specific parameters.

In particular the Distensibility index (DI), minimum cross-sectional area (CSA), minimal diameter (Dmin), and intra-balloon pressure (IBP) were measured. Intraoperative procedural feasibility and EndoFLIP™-related intraoperative adverse events served as the secondary outcome. The study was approved by the local ethics committee (Ethikkommission Land Salzburg: 1005/2023) and was conducted in accordance with the Declaration of Helsinki (as revised in 2013). Electronic data collection was performed in the individual patient file. A two-tailed Welch’s *t*-test for independent samples was conducted using Microsoft Excel (version 365, Microsoft, Redmond, Washington, DC, USA). For each group, the mean and sample variance were calculated, which were then used to compute the test statistic and the degrees of freedom according to the Welch–Satterthwaite formula. Additionally, a 95% confidence interval for the mean difference was calculated to quantify the range within which the true difference is expected to lie with high certainty. The significance of the observed difference was evaluated based on the two-tailed *p*-value, with *p*-values below 0.05 (α = 0.05) considered statistically significant. Due to the pilot study nature and the study design, no adjustment for multiple comparisons of EndoFLIP^TM^ parameters was performed.

## 3. Results

Intraoperative EndoFLIP^TM^ evaluation of the lower esophageal sphincter in sleeve gastrectomy and gastric bypass was successfully performed using the 8 cm EndoFLIP^TM^ catheter (EF-325) in all patients. No EndoFLIP^TM^ -related adverse events occurred on either the gastric sleeve or the anastomosis. An endoscopic air leak test was performed in all patients immediately after the EndoFLIP^TM^ test to exclude any insufficiency or bleeding of staple lines or sutures. None of the patients was complaining about dysphagia preoperatively. In the sleeve gastrectomy group, none of the patients reported of heartburn, whereas in the bypass group patients with heartburn history were eligible for RYGB. However, only one patient had clinically relevant reflux esophagitis of Los Angeles Grade B. Preoperative endoscopy was performed in all patients to rule malignancy, and a barium swallow test was performed to detect major motility disorders or delayed gastric emptying. Preoperative BMI did not differ significantly between the two groups (46.4 kg/m^2^ vs. 44.4 kg/m^2^, *p* = 0.440). Detailed patient demographics and perioperative parameters are listed in Table 1, whereas a detailed presentation of the intraoperative EndoFLIP^TM^ parameters is given in Table 2 for the sleeve gastrectomy group and in Table 3 for the gastric bypass group.

## 4. Discussion

This study demonstrates that the EndoFLIP^TM^ can be applied safely in a variety of internationally established MBS procedures. Moreover, we could demonstrate that functional parameters change significantly after sleeve gastrectomy. In contrast, although the surgical approach at the cardia, the angle of HIS, and the greater curvature was comparable, gastric bypass surgery did not induce any significant functional alterations of the lower esophageal sphincter. It is of note that EndoFLIP™ measurement data given in the literature exhibit some variability. This might be attributed to differences in filling volume of the balloon, patient positioning, timing of measurement during breathing, or the presence of pneumoperitoneum.

To minimize this range, we standardized the measurements based on previously published recommendations. As pneumoperitoneum and patient positioning might have the greatest bias regarding EndoFLIP^TM^ measurements [16], we timed our measuring process after a 30 s filling and calibration period without pneumoperitoneum and with an anti-Trendelenburg position. In accordance with the current literature regarding the surgical use of EndoFLIP^TM^, we opted for the use of a 8 cm balloon size [17]. The first assessment was performed immediately after intubation and was considered as the preoperative measurement. This approach was particularly justified by studies from Su B. et al. [16], who demonstrated that the type of anesthesia or sedation has no significant effect on distensibility results. This study clearly demonstrates that no intraoperative EndoFLIP^TM^-related adverse events occurred, especially regarding manipulation of the newly created pouch or gastric sleeve.

Currently, there are only sporadic publications on the use of EndoFLIP^TM^ in patients with MBS. EndoFLIP^TM^ is advocated as a measure in the context of postoperative adverse events, but distensibility values of the LES are not described in these publications [18,19,20].

With the present study, we were able to obtain intraoperative physiological data from the lower esophageal sphincter during sleeve gastrectomy and gastric bypass. We demonstrated that a significant alteration of the anti-reflux barrier occurs immediately intraoperatively. Specifically, distensibility, diameter, and opening area at the lower esophageal sphincter were found to be significantly increased directly after sleeve formation (Table 1) whereas gastric pouch formation did not lead to significant functional changes (Table 2).

Based on intraoperative findings following fundoplication, elevated Distensibility index values have been shown to correlate with a higher risk of reflux recurrence [21]. Therefore, sleeve gastrectomy appears to cause a direct and intraoperatively detectable compromise of the anti-reflux competence of the lower esophageal sphincter. This is in line with previously published data by Reynolds JL et al. [22] and Magyar CTJ et al. [23] who were able to demonstrate a significant increase in DI after sleeve formation with preoperative measurements of 1.2 mm^2^/mmHg and 1.4 mm^2^/mmHg, compared to postoperative measurements of 2.2 mm^2^/mmHg (*p* = 0.017) and 2.9 mm^2^/mmHg (*p* = 0.046), respectively. These data match the DI measured in our sleeve gastrectomy patient cohort (2.1 mm^2^/mmHg vs. 2.9 mm^2^/mmHg, 95% CI: −1.6 to −0.14, *p* = 0.028).

Despite sleeve gastrectomy being the most frequently performed metabolic bariatric operation, the development of gastroesophageal reflux disease, which represents the most common complication after SG, remains incompletely understood. The existing evidence is conflicting, and the mechanisms involved are regarded as multifactorial and complex. In the absence of an evident anatomical abnormalities such as stenosis or kinking, management is often limited to observation until gastroesophageal reflux becomes clinically apparent during follow-up [24]. The alteration in distensibility may potentially unravel a first functional insight in understanding the refluxogenic component of sleeve gastrectomy, since de novo reflux and Barrett esophagus are known to associated with SG [25,26].

In contrast, no significant difference in distensibility was observed in the gastric bypass cohort despite a similar surgical technique at the angle of HIS (4.0 mm^2^/mmHg vs. 4.0 mm^2^/Hg, 95% CI: −1.8 to 1.8, *p* = 0.988). This suggests that it is not only manipulation around the lower esophageal sphincter itself and the angle of HIS that influences LES function. A key difference in gastric bypass compared to sleeve gastrectomy is the vagotomy when entering the bursa at the incisura angularis during pouch formation, as well as the reconstruction, since the gastrojejunostomy represents a downstream low-pressure zone in contrast to the potentially narrow incisura angularis or pylorus in sleeve gastrectomy.

To the best of our knowledge, this is the first study providing intraoperative EndoFLIP^TM^ baseline data in gastric sleeve gastrectomy and gastric bypass.

### Limitations

The definition of normative values has to be further clarified by evaluating the clinical follow-up and larger case numbers.

Further investigations of long term follow-up data are of great interest to address the correlation of EndoFLIP^TM^ measurements with postoperative development of complication such as weight regain, reflux symptoms, or development of strictures.

Due to the small sample size, the pilot design of the study and demographic variables such as gender, age, height, and weight could still influence data analysis and the possibility of a type II error. This limitation should be easily overcome by conducting further studies with a larger caseload.

## 5. Conclusions

Sleeve gastrectomy immediately and significantly influences the LES and increases the opening area whereas gastric bypass surgery appears not to influence LES distensibility or opening diameters. Intraoperative standardized EndoFLIP^TM^ measurements are feasible and safe and add additional real-time information during MBS.

## Figures and Tables

**Table 1:** Patient demographics and perioperative parameters.

Patient Demographics	
**Patients (*n*)**	40
Female	31
Male	9
Age (years)	40 (23–65)
BMI (kg/m^2^)	45.2 (35–68)
ASA classification	3 (2–3)
Comorbidities	
Diabetes mellitus	7 (18)
Obstructive sleep apnea	12 (30)
Bronchial asthma	3 (7.5)
NAFLD	2 (5)
**Procedure *n* (%)**	
Sleeve gastrectomy	17 (42)
OAGB	15 (38)
RYGB	8 (20)
Hospital stay after surgery (days)	5 (4–16)
EndoFLIP^TM^-related adverse events n (%)	0 (0)

Abbreviations: BMI (body mass index); ASA (American Society of Anesthesiologists); ERD (erosive reflux disease); NAFLD (non-alcoholic fatty liver disease); OAGB (one-anastomosis gastric bypass); RYGB (Roux-en-Y gastric bypass). Data are presented as mean (range) or total number (percentage).

**Table 2:** Intraoperative EndoFLIP^TM^ results at the LES before and after sleeve gastrectomy.

LES Measurements	Before	After	95% CI	*p*-Value
Dmin	12.0 (±1.2)	13.9 (±2.8)	−3.6 to −0.2	0.028
DI	2.1 (±0.5)	2.9 (±1.3)	−1.6 to −0.14	0.023
IBP	56.3 (±7.6)	55.6 (±12.1)	−8.0 to 9.4	0.863
CSA	109.6 (±28.1)	157.8 (±56.0)	−84.2 to −12.3	0.011

Abbreviations: LES (lower esophageal sphincter, distance in cm); DI (Distensibility index in mm^2^/mmHg); Dmin (minimal diameter in mm); IBP (intra-balloon pressure in mmHg); CSA (minimum cross-sectional area in mm^2^); CI (confidence interval). Data are presented as mean ± standard deviation and 95% confidence intervals are given for group differences. The *p*-values refer to two-sided tests.

**Table 3:** Intraoperative EndoFLIP^TM^ results at the LES before and after gastric bypass.

LES Measurements	Before	After	95% CI	*p*-Value
Dmin	14.1 (±3.6)	15.4 (±2.6)	−4.5 to 1.9	0.389
DI	4.0 (±2.0)	4.0 (±2.0)	−1.8 to 1.8	0.988
IBP	46.7 (±12.6)	51.0 (±12.2)	−15.6 to 7.0	0.425
CSA	166.8 (±73.3)	191.4 (±65.4)	−89.8 to 40.5	0.422

Abbreviations: LES (lower esophageal sphincter, distance in cm); DI (Distensibility index in mm^2^/mmHg); Dmin (minimal diameter in mm); IBP (intra-balloon pressure in mmHg); CSA (minimum cross-sectional area in mm^2^); CI (confidence interval). Data are presented as mean ± standard deviation and 95% confidence intervals are given for group differences. The *p*-values refer to two-sided tests.

## Data Availability

The data presented in this study are available on request from the corresponding author. The data are not publicly available due to privacy and ethical restrictions.

## Abbreviations

The following abbreviations are used in this manuscript:

ASA	American Society of Anesthesiologists
BMI	Body mass index
CSA	Cross-sectional area
DI	Distensibility index
Dmin	Minimal diameter
GB	Gastric bypass
IBP	Intra-balloon pressure
LES	Lower esophageal sphincter
MBS	Metabolic baria
OAGB	One-anastomosis gastric bypass
RYGB	Roux-en-Y gastric bypass
SG	Sleeve gastrectomy
UGIT	Upper gastrointestinal tract
